# Prospective study on human fecal carriage of *Enterobacteriaceae* possessing *mcr-1* and *mcr-2* genes in a regional hospital in Hong Kong

**DOI:** 10.1186/s12879-018-2987-y

**Published:** 2018-02-13

**Authors:** Wai-Sing Chan, Chun-Hang Au, Dona N. Ho, Tsun-Leung Chan, Edmond Shiu-Kwan Ma, Bone Siu-Fai Tang

**Affiliations:** Department of Pathology, 1/F, Li Shu Fan Block, Hong Kong Sanatorium & Hospital, 2 Village Road, Happy Valley, Hong Kong

**Keywords:** Colistin resistance, Fecal carriage, Hong Kong, *mcr-1* and *mcr-2*

## Abstract

**Background:**

Human fecal carriage of *Enterobacteriaceae* possessing mobilized colistin resistance genes (*mcr-1* and *mcr-2*) remains obscure in Hong Kong. As part of routine surveillance on emerging antibiotic resistance, we conducted a prospective study on this topic in a regional hospital in Hong Kong.

**Methods:**

From October 31 to November 25, 2016, all fecal specimens submitted for routine analysis were included in this surveillance study. These comprised 672 consecutive routine fecal specimens collected from 616 individuals. Fecal specimens were screened for colistin-resistant *Enterobacteriaceae* by culture-based method, and the presence of *mcr-1* and *mcr-2* genes in resistant isolates was identified by polymerase chain reaction and Sanger sequencing. Whole genome sequencing (WGS) of *mcr-1-*possessing *Escherichia coli* strains was facilitated using Illumina® MiSeq® followed by sequence analysis with appropriate bioinformatics tools.

**Results:**

Fourteen *mcr-1-*positive *E. coli* strains were isolated from 14 separate individuals (2.08% of total fecal specimens), with 9 of them being asymptomatic, healthy clients coming for health assessment. No *mcr-2*-possessing *Enterobacteriaceae* was identified. Colistin minimum inhibitory concentrations of these *mcr-1-*positive isolates ranged from 2 to 4 μg/mL. All these isolates were susceptible to carbapenems with 2 being extended spectrum β-lactamase producers. WGS data revealed that these isolates belonged to at least 12 different sequence types (STs) and possessed diversified plasmid replicons, virulence and acquired antibiotic resistance genes. Further study on an *E. coli* ST201 strain (Pasteur scheme) revealed coexistence of 47,818-bp IncP-1 and 33,309-bp IncX4 types of *mcr-1* plasmids, which was a combination of stability and high transmissibility.

**Conclusions:**

To the best of our knowledge, this is the first study on human fecal carriage of *Enterobacteriaceae* possessing *mcr-1* and *mcr-2* genes in Hong Kong. Our data further revealed asymptomatic carriage of *mcr-1-*possessing *Enterobacteriaceae* by both patients and healthy individuals. This is alarming considering wide diversity and high transmissibility of *mcr-1* plasmids, which potentially facilitate emergence of pan-drug-resistant bacteria in future infection. This also highlights the importance of surveillance on emerging antibiotic resistance, especially for patients under intensive care.

## Background

Recent reports on plasmid-mediated colistin resistance of *Enterobacteriaceae* from research groups worldwide have heralded concerns about emergence of ‘superbugs’ which are resistant to this last resort of treatment [1–3]. With broad range of compatible plasmids and bacterial hosts [4], mobilized colistin resistance gene (*mcr-1*) has successfully disseminated globally. The presence of *mcr-1-*carrying *Enterobacteriaceae* in food animals worldwide [1, 5, 6] further facilitates transmission of *mcr-1* to human. *Escherichia coli* has been the major bacterial host of *mcr-1* gene, and recent study has unraveled significant geographical clustering with regional spread of IncHI2 and IncI2 types of *mcr-1* plasmids in Europe and Asia, respectively [7].

In Hong Kong, Wong and coworkers reported a *mcr-1* positive rate of 0.4% among 1,324 *Enterobacteriaceae* isolates from patients, which included 2 asymptomatic carriers [8]. At present, no prevalence data on human fecal carriage of *mcr-1- and mcr-2*-possessing *Enterobacteriaceae* has been documented in our region. As a routine surveillance on emerging antibiotic resistance, we conducted a prospective study on this topic in a regional hospital in Hong Kong.

## Methods

### Collection of human fecal specimens

The goal of this surveillance study was to have a gross picture on (1) fecal carriage of *mcr-1-* and *mcr-2-*harboring *Enterobacteriaceae* among patients/ clients using our service, (2) characteristics of the carriers and (3) phenotypic and molecular traits of the positive strains. This information is important for infection control and risk assessment. From October 31 to November 25, 2016, all fecal specimens submitted for routine analysis were included in this surveillance study. These comprised 672 consecutive routine fecal specimens collected from 616 individuals, including 79 fecal specimens from 67 outpatients, 171 fecal specimens from 144 inpatients and 422 fecal specimens from 418 asymptomatic clients coming for health assessment. Forty out of 144 inpatients had been screened for vancomycin-resistant enterococci (VRE), which is routinely performed for inpatients who have been hospitalized in/ outside Hong Kong, underwent VRE screening in other hospitals, undergone surgical operation overseas or staying in a nursing home within past 6 months. After routine testing, leftover fecal specimens were used for surveillance screening.

### Screening of colistin-resistant *Enterobacteriaceae* in fecal specimens

The concept of Blackburn and coworkers’ method for screening carbapenem-resistant bacteria was adopted [9], with some modifications. Briefly, fecal specimens with approximate sizes of 10 μL inoculation loop were homogenized in 500 μL sterile, double-deionized water and centrifuged at 1,500 rpm for 30 s. An aliquot of 10 μL supernatant from each sample was inoculated on chromID® CPS® Elite agar (bioMérieux, Marcy, I’Etoile, France) and a piece of polymyxin B disc (PB300, Oxoid, Basingstoke, Hampshire, England) was applied on the area of inoculation, followed by overnight incubation at 35 ^o^C in ambient air. Bacterial growth around polymyxin B disc, namely inside the inhibition zone of approximately 11 mm in diameter (taking the Clinical and Laboratory Standards Institute (CLSI) guidelines’ interpretative criteria of polymyxin B for *Pseudomonas aeruginosa* as reference), was subjected to polymerase chain reaction (PCR) targeting *mcr-1* and *mcr-2* genes.

### Colony PCR targeting *mcr-1* and *mcr-2* genes

Polymyxin B-resistant bacterial colonies from each sample were homogenized in 20 μL sterile, double-deionized water. A 1 μL aliquot of this suspension was added to PCR cocktail comprising 20 μL Platinum® PCR Supermix (Invitrogen, Carlsbad, CA) and 1 μL of each 10 μM primer (*mcr-1* forward primer, 5’-ATGATGCAGCATACTTCTGTG-3’ [10] and reverse primer, 5’-CCCAAACCAATGATACGCAT-3’ [8], corresponding to amplicon size of 398 bp; *mcr-2* forward primer, 5’-TGTTGCTTGTGCCGATTGGA-3’ and reverse primer, 5’-AGATGGTATTGTTGGTTGCTG-3’ [2], corresponding to amplicon size of 567 bp). PCR was performed using GeneAmp® PCR System 9700 (Applied Biosystems, Foster City, CA, USA) with initial cell lysis and denaturation at 95 ^o^C for 10 min, three-step cycling for 40 times (95 ^o^C for 10 s, 55 ^o^C for 30 s and 72 ^o^C for 30 s) and final extension at 72 ^o^C for 5 min. The amplicons were electrophoresized in 2% agarose gel.

### Bacterial identification of PCR-positive isolates

PCR-positive colony suspensions were subcultured on CPS agar and incubated at 35 ^o^C overnight in ambient air. Recovered bacterial colonies were identified by matrix-assisted laser desorption/ionization-time of flight mass spectrometry (MALDI-TOF MS, Microflex LT, Bruker Daltonik, Bremen, Germany). Briefly, minute amount of bacterial colony was smeared on target plate and covered with 1 μL of 10 mg/mL matrix solution (α-cyano-4-hydroxycinnamic acid, Bruker Daltonik, Bremen, Germany). The dried samples were subjected to MS measurement after successful calibration with Bacterial Test Standard (Bruker Daltonik, Bremen, Germany). Spectra were obtained with an accelerating voltage of 20 kV in linear mode with m/z range of 2,000 to 20,000 Da, followed by analysis with MALDI Biotyper version 3.0 and reference library version 3.1.2.0 (Bruker Daltonik, Bremen, Germany).

### Antibiotic susceptibility testing of PCR-positive isolates

Antibiotic susceptibilities were determined using Kirby-Bauer disk diffusion method with reference to CLSI guidelines. Minimum inhibitory concentration (MIC) of colistin was determined using broth microdilution (BMD) (SensiTest Colistin, Liofilchem® Diagnostics, L’ Aquila, Italy). Susceptibility results of VITEK® 2 AST cards (AST-N338, bioMérieux, Marcy, I’Etoile, France) and polymyxin B Etest strips (bioMérieux, Marcy, I’Etoile, France) served as additional reference.

### Sanger sequencing of full *mcr-1* genes

Total nucleic acid of PCR-positive isolates was extracted using NucliSENS® easyMAG® automated system (bioMérieux, Marcy, I’Etoile, France) and full *mcr-1* genes were amplified using primers published by Ye and co-workers [10]. Amplicons were purified enzymatically with ExoSAP-IT® (Affymetrix, Santa Clara, CA), followed by cycle sequencing using BigDye® Terminator v.1.1 Cycle Sequencing Kit (Applied Biosystems, Foster City, CA, USA) and purification of sequencing products using BigDye® XTerminator™ Purification Kit (Applied Biosystems, Foster City, CA, USA). Purified sequencing products were analyzed using 3130*xl* Genetic Analyzer/ 3730 DNA Analyzer (Applied Biosystems, Foster City, CA, USA).

### Whole genome sequencing (WGS) of *mcr-1-*possessing *E. coli* strains

Briefly, DNA concentrations of total nucleic acid extracts were measured using Quant-it™ PicoGreen® dsDNA Assay Kit via Qubit® 2.0 Fluorometer (Invitrogen, Carlsbad, CA). DNA concentrations were adjusted to 0.2 ng/μL, followed by tagmentation and limited-cycle amplification using Nextera XT DNA Library Prep Kit (Illumina, San Diego, CA, USA). Indexed libraries were purified using Agencourt® AMPure® XP (Beckman Coulter, Beverly, MA, USA) and analyzed by 2 × 300 bp paired-end sequencing on MiSeq platform using MiSeq v3 Reagent Kit (Illumina, San Diego, CA, USA).

### Bioinformatics analysis of WGS data

WGS short reads were assembled into contigs using SPAdes version 3.10.1 [11]. *E. coli* sequence types (STs) were determined using MLST databases of The University of Warwick [12] and PubMLST [13]. Acquired resistance genes were identified using ResFinder 2.1 [14]. Virulence genes were detected using VirulenceFinder 1.5 [15]. Alignment of contigs to appropriate reference sequences was performed using Mauve version 2.4.0 [16] followed by post-assembly gap filling by PCR and Sanger sequencing. Prokka version 1.11 [17] was used for plasmid annotation with manual editing. Plasmid replicons and insertion sequences were identified using PlasmidFinder 1.3 [18] and IS finder [19]. Plasmid genetic maps were generated using SnapGene® Viewer 4.1. Sequence comparisons between plasmids were generated using BLAST Ring Image Generator (BRIG) [20].

## Results and Discussion

### Fecal carriage of *mcr-1-/ mcr-2-*possessing *Enterobacteriaceae*

Seventy nine out of 672 fecal specimens revealed bacterial growth inside the inhibition zone of polymyxin B disc and subjected to colony PCR, with 14 specimens being *mcr-1-*positive (2.08%) and none being *mcr-2-*positive. Results of MALDI-TOF MS showed that all 14 isolates were *E. coli* (top identification scores ranged from 2.271 to 2.526) which were consistent with the appearance of bacterial colonies on CPS agars (red-colored). These *E. coli* strains were isolated from 14 separate individuals including 3 inpatients, 2 outpatients and 9 asymptomatic clients coming for health assessment, with age from 1 to 58 years (Table 1). Regarding available clinical/ epidemiological information of these *mcr-1* carriers, Subject 1 and 10 had received antibiotic treatment other than colistin prior to fecal specimen collection. For others, none had been prescribed with antibiotics in our hospital. We could not access any information on antibiotic usage outside our hospital. Subject 3 had been hospitalized in another hospital within 6 months prior to admission. None of these *mcr-1-*possessing *E. coli* strains caused diseases in their human hosts. In general, no apparent correlation was observed between *mcr-1* fecal carriage and the available clinical/ epidemiological information.

**Table 1:** Subject details and antibiotic susceptibilities of the 14 *mcr-1-*positive *E. coli* strains

Subject	Age/Year	Subject type^a^	ESBL^b^ producer?	Carbapenem susceptible?	PB MIC^c^ (μg/mL)	PE MIC^d^ (μg/mL)	PE MIC^e^ (μg/mL)	Non-susceptible antibiotics^g^ (based on CLSI guidelines)
1^f^	45	OP	No	Yes	8	4	8	AM^R^, CF^I^, SXT^R^
2	54	HA	No	Yes	8	2	8	SXT^R^
3	42	IP	No	Yes	8	4	8	CF^I^, MI^I^, SXT^R^
4	54	HA	No	Yes	12	4	8	AM^R^, AMC^I^, AN^I^, MI^R^, SXT^R^
5	2	IP	No	Yes	4	4	4	AM^R^, AMC^R^, AN^R^, CF^R^, CIP^R^, CN^R^, CXM^R^, LEV^I^, SXT^I^, TIM^R^, TOB^R^, TZP^R^
6	45	HA	No	Yes	8	4	8	AM^R^, AMC^I^, CIP^R^, CN^R^, CXM^I^, LEV^R^, SXT^R^, TIM^I^, TOB^R^
7	43	HA	No	Yes	8	4	4	AM^R^, AMC^I^, CF^R^, CIP^I^, CXM^I^, LEV^I^, MI^I^, SXT^R^, TIM^I^, TZP^R^
8	31	HA	No	Yes	12	4	> 16	All susceptible
9	52	HA	No	Yes	4	2	4	CF^I^, CXM^I^
10^f^	1	IP	No	Yes	6	4	8	AM^R^, AN^I^, CF^R^, CXM^I^, MI^I^, TOB^I^
11	58	HA	No	Yes	8	4	8	AM^R^, SXT^R^
12	7	OP	Yes	Yes	12	4	4	AM^R^, AMC^R^, CAZ^R^, CF^R^, CTX^R^, CXM^R^, FEP^R^, SXT^R^
13	49	HA	Yes	Yes	8	4	4	AM^R^, AMC^R^, AN^I^, CAZ^R^, CF^R^, CIP^R^, CN^R^, CTX^R^, CXM^R^, FEP^R^, LEV^I^, SXT^R^, TOB^R^
14	55	HA	No	Yes	6	2	2	AM^R^, CF^R^, CIP^I^, CXM^I^, MI^I^, SXT^R^

^a^OP outpatients, HA clients coming for health assessment, IP inpatients

^b^ESBL, extended-spectrum β-lactamase

^c^PB MIC, Polymyxin B minimum inhibitory concentration by Etest

^d^PE MIC, Polymyxin E (colistin) minimum inhibitory concentration by broth microdilution (SensiTest Colistin)

^e^PE MIC, Polymyxin E (colistin) minimum inhibitory concentration by Vitek® 2 AST N-338

^f^The subjects had received antibiotic treatment other than colistin prior to fecal specimen collection

^g^*I* intermediate, *R* resistant, *AM* ampicillin, *AMC* amoxicillin-clavulanate, *AN* amikacin, *CAZ* ceftazidime, *CF* cephalothin, *CIP* ciprofloxacin, *CN* gentamicin, *CTX* cefotaxime, *CXM* cefuroxime, *FEP* cefepime, *LEV* levofloxacin, *MI* minocycline, *SXT* trimethoprim/sulfamethoxazole, *TIM* ticarcillin-clavulanate, *TOB* tobramycin, *TZP* piperacillin-tazobactam

The fecal carriage data further revealed asymptomatic carriage of *mcr-1-*possessing *Enterobacteriaceae* by both patients and healthy individuals. The *mcr-1* positive rate estimated in this study (2.08%) was much higher than that reported by another local research group (0.4%) [8], which implies the prevalence was higher than expected in Hong Kong. This difference in *mcr-1* positive rate might be explained by different screening targets adopted, with the study by Wong and coworkers focusing on clinical isolates whereas our study screening fecal specimens. As fecal specimens normally contain numerous strains of *Enterobacteriaceae* per sample, the chance of isolating *mcr-1-*harboring strains might be increased. Compared with human fecal carriage data of other regions, the *mcr-1* positive rate estimated in this study was lower than that of mainland China (4.9 - 6.2%) [21, 22] and higher than that of several European countries (Switzerland, 0% [23]; Western France, 0% [24]; The Netherlands, 0.35% [25]). This is not surprising considering high prevalence of *mcr-1-*harboring *Enterobacteriaceae* among livestock animals in mainland China [26], which is a major supplier of food animals in Hong Kong. On the other hand, no *mcr-2-*carrying *Enterobacteriaceae* was isolated. In another large-scale study in Japan, *mcr-2* gene was not detected among 9,306 *E. coli* strains isolated from healthy animals [27]. Considering these prevalence data together with reports of zero human fecal carriage from various European countries [23–25], it appears that *mcr-2-*carrying *Enterobacteriaceae* has not been disseminated globally at present.

### Antibiotic susceptibility of *mcr-1-*positive isolates

Antibiotic susceptibility results are summarized in Table 1. Colistin MICs ranged from 2 to 4 μg/mL by BMD and 2 to > 16 μg/mL by VITEK® 2 AST. The range of polymyxin B MICs was 4 to 12 μg/mL by Etest. The *mcr-1*-positive isolates displayed different patterns of susceptibility to a panel of 19 antibiotics for *Enterobacteriaceae*. With exception of Subject 8, all other isolates were intermediate or resistant to at least 1 antimicrobial agent, including penicillins (ampicillin, 71.4%), β-lactam/β-lactamase inhibitor combinations (amoxicillin-clavulanate, 50%; piperacillin/tazobactam, 14.3%; ticarcillin-clavulanate, 21.4%), cephems (cefepime, 14.3%; cefotaxime, 14.3%; ceftazidime, 14.3%; cefuroxime, 57.1%; cephalothin, 64.3%), aminoglycosides (amikacin, 28.6%; gentamicin, 21.4%; tobramycin, 28.6%), tetracyclines (minocycline, 35.7%), quinolones (ciprofloxacin, 35.7%; levofloxacin, 28.6%) and folate pathway inhibitors (trimethoprim/sulfamethoxazole, 78.6%). All these isolates were susceptible to carbapenems with 2 being extended-spectrum β-lactamase (ESBL) producers.

The range of colistin MICs in this study was 2 to 4 μg/mL by BMD, which appeared to be narrower than that of *mcr-1-*positive *E. coli* isolates causing human diseases (4 to 16 μg/mL by BMD) reported by various research groups [28–31]. Colistin MICs determined by VITEK® 2 AST were consistent with that of BMD for 35.7% of the isolates, whereas MICs were 2- to > 4-fold higher than that of BMD for the remainder. Albeit underestimation of colistin MIC was not observed in this study, the suitability of using VITEK® 2 AST for colistin MIC determination awaits further evaluation due to its high very major error rate (36%) revealed by a recent study [32]. In addition, as 21.4% of the isolates displayed colistin MICs lower than the resistant breakpoint (> 2 μg/mL) published by The European Committee on Antimicrobial Susceptibility Testing (EUCAST), we echo the suggestion of using a lower susceptible breakpoint of < 1 μg/mL by Chew and coworkers [32] for improving detection of *mcr-1-*possessing *Enterobacteriaceae.* On the other hand, it was noticeable that more than half of the isolates (57.1%) displayed a ‘baseline’ insusceptibility to 5 or more antibiotics, and 2 of which were ESBL producers. The choice of usable antibiotics may be further limited for these isolates upon acquisition of antibiotic resistance genetic elements, for instance, carbapenemase genes.

### Molecular characteristics of *mcr-1-*positive isolates

The results are summarized in Table 2.

**Table 2:** Molecular characteristics of the 14 *mcr-1-*positive *E. coli* strains

Subject	Sequence Type	Plasmid Replicons	Virulence Genes	Acquired Resistance Genes
1	442 (Achtman) Unknown (Pasteur)	IncI1	*cma, iroN, iss, lpfA*	*aadA1, aadA2, bla*_*TEM-1B*_ *, dfrA12,* ***mcr-1*** *, QnrS1, sul3, tet(A),*
2	Unknown (Achtman) 201 (Pasteur)	IncX4, IncP1	*astA, iss, lpfA, mchF, tsh*	*aadA1, aadA2, dfrA12,* ***mcr-1*** **(‘R’ at nt. 1263)**, *sul3, tet(A)*
3	88 (Achtman) 66 (Pasteur)	Not detected	*cma, iroN, iss, lpfA*	*aadA5,* ***mcr-1*** *, mph(A), strA, strB, sul1*
4	Unknown (Achtman) Unknown (Pasteur)	IncX4	*iss*	*aadA1, aadA2, bla*_*TEM-1B*_*,* ***mcr-1*** **(27C>T)**, *sul3, tet(B)*
5	Unknown (Achtman) 1716 (Pasteur)	IncI1	*astA*	*aac(6’)Ib-cr, aadA5, ARR-3, dfrA17, bla*_*OXA-1*_ *,* ***mcr-1*** *, oqxA, oqxB, QnrS2*
6	155 (Achtman) 21 (Pasteur)	IncN	*lpfA*	*aadA1, aadA2, aph(3’)-Ia, bla*_*TEM-1B*_ *, dfrA12,* ***mcr-1*** *, mph(A), oqxA, oqxB, sul2, sul3*
7	10 (Achtman) 2 (Pasteur)	IncN	Not detected	*aadA1, aadA2, aph(3’)-Ia, bla*_*TEM-1B*_ *, dfrA12,* ***mcr-1*** *, mph(A), sul2, sul3*
8	34 (Achtman) 638 (Pasteur)	Not detected	Not detected	***mcr-1***
9	226 (Achtman) 486 (Pasteur)	IncR, IncX4	Not detected	***mcr-1*** *, strA, strB, tet(B)*
10	5995 (Achtman) Unknown (Pasteur)	IncQ1	Not detected	*aph(3’)-Ia, bla*_*TEM-1B*_ *,* ***mcr-1*** *, strA, strB*
11	95 (Achtman) 1 (Pasteur)	IncI1, IncI2, IncQ1	*iss, sfaS*	*aph(3’)-Ia, bla*_*TEM-1B*_ *, dfrA17,* ***mcr-1*** *, oqxA, oqxB, strA, strB, sul2, tet(B)*
12	48 (Achtman) Unknown (Pasteur)	Not detected	Not detected	*aadA2, bla*_CTX-M-9_-like gene, *dfrA12,* ***mcr-1****, sul3*
13	48 (Achtman) Unknown (Pasteur)	IncN, IncHI2	Not detected	*aadA1, bla*_CTX-M-9_-like gene, *dfrA12, fosA3,* ***mcr-1****, QnrS1, sul1, sul3*
14	746 (Achtman) Unknown (Pasteur)	Not detected	*astA*	*aadA1, aadA2, aph(3’)-Ia, dfrA12,* ***mcr-1*** *, oqxA, oqxB, QnrS1, sul3*

### *mcr-1* gene sequences

The *mcr-1* gene sequences of 12 isolates were 100% identical to that of *E. coli* strain SHP45 (NG_050417), with exception of the isolate from Subject 4 harboring C>T substitution at nucleotide position 27 and the isolate from Subject 2 with mixed nucleotides (R) at position 1263, indicating possible coexistence of ‘wild type’ and 1263G>A variant in the same bacterium. Both of these nucleotide substitutions were silent mutations.

### *E. coli* sequence types

Thirteen isolates were assigned to 12 *E. coli* STs by Achtman or Pasteur scheme, including ST442 (Achtman), ST201 (Pasteur), ST88 (Achtman)/ ST66 (Pasteur), ST1716 (Pasteur), ST155 (Achtman)/ ST21 (Pasteur), ST10 (Achtman)/ ST2 (Pasteur), ST34 (Achtman)/ ST638 (Pasteur), ST226 (Achtman)/ ST486 (Pasteur), ST5995 (Achtman), ST95 (Achtman)/ ST1 (Pasteur), ST746 (Achtman) and ST48 (Achtman, 2 ESBL-producing strains). The strain from Subject 4 could not be assigned to currently known ST by both Achtman and Pasteur schemes. Generally, the STs of these strains were diversified. Albeit both the strains from Subject 12 and 13 were assigned to ST48, their antibiotic susceptibility patterns, genetic context and Pasteur ST profiles were different, which appeared that they were divergent over years rather than a short period of time. Among these STs, ST10, ST34 and ST48 have been reported to possess *mcr-1* gene [33], with ST10 being the most common among *mcr-1-*possessing *E. coli* [7]. On the other hand, ST95, ST155, ST226, ST442 and ST746 have been associated with human infection [34–38].

### Virulence genes

Virulence genes were detected in 8 isolates, including *astA* (21.4%), which encodes EAST-1 heat-stable toxin; *cma* (14.3%), which encodes colicin-M; *iroN* (21.4%), which encodes enterobactinsiderophore receptor protein; *iss* (35.7%), which correlates with increased serum survival of *E. coli*; *lpfA* (28.6%), which encodes long polar fimbriae; *mchF* (7.1%), which encodes ABC transporter protein; *sfaS* (7.1%), which encodes S-fimbrial adhesion protein and *tsh* (7.1%), which encodes temperature-sensitive hemagglutinin. These genes are associated with mild virulence of *E. coli*.

### Plasmid incompatibility groups

Eight plasmid replicon types were identified in 10 isolates, with IncI1 (21.4%), IncX4 (21.4%) and IncN (21.4%) being the predominant incompatibility groups, followed by IncQ1 (14.3%), IncP1 (7.1%), IncR (7.1%), IncI2 (7.1%) and IncHI2 (7.1%). Previous study has shown that IncX4, IncI2 and IncHI2 incompatibility groups comprised 90.4% of identified *mcr-1* plasmids [7]. Significant geographical clustering was observed with regional spread of IncI2 and IncHI2 types of *mcr-1* plasmids in Asia and Europe, respectively, while there was no significant difference between distribution of these major replicon types from different sources of isolation [7]. In this study, albeit further work is needed to confirm the genomic locations of *mcr-1* genes, plasmid replicon types were diversified and not confined to IncI2, which was detected in a single isolate from Subject 11. Further study with larger cohort of isolates from different sources is needed to determine local characteristics of *mcr-1* plasmid incompatibility groups.

### Acquired antibiotic resistance genes

With exception of *mcr-1*, acquired antibiotic resistance genes were identified in 13 out of 14 isolates. Aminoglycoside resistance-related genes were the most commonly observed, including *aadA1* (50%), *aadA2* (50%), *aadA5* (14.3%), *aph(3’)-Ia* (35.7%), *strA* (28.6%) and *strB* (28.6%). This was followed by genes related to sulphonamide resistance (*sul1*, 14.3%; *sul2*, 21.4%; *sul3*, 57.1%), quinolone resistance (*aac(6’)Ib-cr*, 7.1%; *oqxA*, 28.6%; *oqxB*, 28.6%; *QnrS1*, 21.4%; *QnrS2*, 7.1%), β-lactam resistance (*bla*_OXA-1_, 7.1%; *bla*_TEM-1B_, 42.9%; *bla*_CTX-M-9_-like, 14.3%), trimethoprim resistance (*dfrA12*, 50%; *dfrA17*, 14.3%), tetracycline resistance (*tet(A)*, 14.3%; *tet(B)*, 21.4%), macrolide resistance (*mph(A)*, 21.4%), rifampicin resistance (*AAR-3,* 7.1%) as well as fosfomycin resistance (*fosA3*, 7.1%). Carbapenemase genes were not detected in these *mcr-1-*possessing *E. coli* isolates.

### Further study on the isolate harboring 2 *mcr-1* genes

#### Genetic structures of the *mcr-1* plasmids

The *E. coli* strain from Subject 2 possessed both ‘wild type’ and 1263G>A variant of *mcr-1* gene. As a matter of interest, we have further studied the genomic locations of *mcr-1* genes in this isolate. WGS data revealed 2 sets of contigs linking to both ends of *mcr-1* sequence. The first set comprised a 20,329-bp contig upstream and a 25,592-bp contig downstream of *mcr-1*; the second set included a 3,508-bp contig upstream and a 3,608-bp contig downstream of *mcr-1.* These 2 contig combinations best matched to plasmid sequences with GenBank accession numbers KY352406 and KX570748, respectively. Therefore, mapping of contigs was based on these 2 reference sequences.

**Fig. 1:**
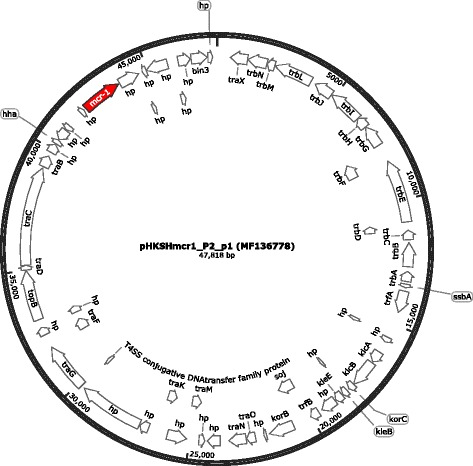
Genetic map and organization of pHKSHmcr1_P2_p1 (GenBank accession No.: MF136778). The circular image was generated with SnapGene® Viewer 4.1. hp, hypothetical protein

**Fig. 2:**
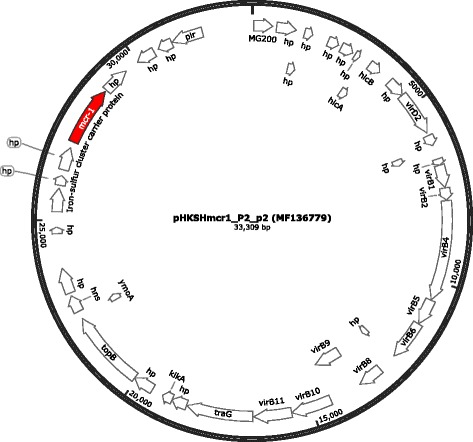
Genetic map and organization of pHKSHmcr1_P2_p2 (GenBank accession No.: MF136779). The circular image was generated with SnapGene® Viewer 4.1. hp, hypothetical protein

The complete *mcr-1* plasmids were 47,818 bp (average depth of coverage = 65.0, GC content = 47.2%) and 33,309 bp (average depth of coverage = 54.8, GC content = 41.8%) in length, possessing single IncP-1 and IncX4 replicon, respectively. No acquired virulence and antibiotic resistance genes were found on both plasmids except *mcr-1*. The IncP-1 plasmid, pHKSHmcr1_P2_p1 (MF136778), possessed 61 coding sequences (CDS), including the 1263G>A *mcr-1* variant, *trfA* (plasmid replication initiation), genes for various cellular functions and 23 hypothetical proteins. No insertion sequence was found on the IncP-1 plasmid. The genetic map is shown in Fig. 1. The IncX4 plasmid, pHKSHmcr1_P2_p2 (MF136779), contained 41 CDS, including the ‘wildtype’ *mcr-1* gene, *pir* (initiation of plasmid DNA replication), genes for various cellular functions and 20 hypothetical proteins. IS26 insertion sequence was present at 3,444 bp upstream of *mcr-1*. The genetic map is shown in Fig. 2.

#### Comparative sequence analysis

We have extracted all nucleotide sequences of IncP-1 and IncX4 types of *mcr-1* plasmids from GenBank (complete sequences only, dated on April 23, 2017) for comparative sequence analysis. Among 104 *mcr-1* plasmid entries from 10 regions, 2 sequences (1.9%) possessed IncP-1 replicons, whereas 30 sequences (28.8%) belonged to IncX4 incompatibility group. Details of these sequences are summarized in Table 3.

**Table 3:** Details of IncP-1 and IncX4 types of *mcr-1* plasmids from GenBank and this study (complete sequences only, retrieved on April 23, 2017)

Accession No.	Plasmid size/bp	Species	Location	Carbapenemase/ESBL genes	Insertion sequences (complete/ partial)
IncP-1 plasmids					
**MF136778 (this study)**	**47,818**	***E. coli***	**Hong Kong**	**No**	**No**
KX377410	57,278	*K. pneumoniae*	China	No	IS26, ISApl1
KY352406	47,824	*S. enterica* subsp. *enterica*	China	No	No
IncX4 plasmids					
**MF136779 (this study)**	**33,309**	***E. coli***	**Hong Kong**	**No**	**IS26**
KX084392	33,298	*E. coli*	China	No	IS26
KU647721	48,350	*E. coli*	China	*bla*_NDM-5_	IS5, IS26, ISAba125, IS3000
KX254343	33,307	*E. coli*	China	No	IS15DI
KX570748	32,751	*E. coli*	China	No	IS26
KX711706	33,309	*E. coli*	China	No	IS26
KX711707	34,997	*E. coli*	China	No	IS26, IS1294
KX711708	34,924	*E. coli*	China	No	ISApl1
KX772777	33,309	*E. coli*	China	No	IS26
KY012276	33,287	*E. coli*	China	No	IS26
KY075652	33,302	*E. coli*	China	No	IS26
KY075653	33,309	*E. coli*	China	No	IS26
KY075655	33,292	*E. coli*	China	No	IS26
KY075660	33,305	*E. coli*	China	No	IS26
KY582848	31,229	*E. coli*	China	No	IS26
KY120363	42,941	*S. enterica* subsp. *enterica*	Taiwan	No	ISSbo1, ISApl1
KY120364	33,308	*S. enterica* subsp. *enterica*	Taiwan	No	IS26
KY471146	47,038	*E. coli*	Korea	No	IS26, IS679, ISApl1
LC227558	33,304	*E. coli*	Japan	No	IS26
CP015977	33,304	*E. coli*	Brazil	No	IS26
CP017246	34,992	*E. coli*	Brazil	No	IS26, IS1294
KU761327	33,287	*K. pneumoniae*	Brazil	No	IS26
KY770023	33,051	*E. coli*	Brazil	No	IS26
KY770024	33,304	*E. coli*	Brazil	No	IS26
KY770025	34,975	*E. coli*	Brazil	No	IS26, IS1294
CP018773	33,305	*E. coli*	USA	No	IS26
KX447768	33,395	*E. coli*	USA	No	IS26
LT838201	33,304	*E. coli*	France	No	IS26
KX129783	34,640	*E. coli*	Switzerland	No	IS2, IS26
KU743383	33,311	*E. coli*	Estonia	No	IS26
KX236309	33,303	*K. pneumoniae*	Italy	No	IS26

**Fig. 3:**
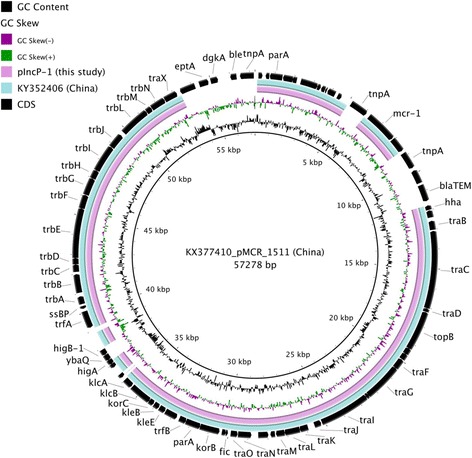
Comparative sequence analysis of IncP-1 type of *mcr-1* plasmids. The circular image was generated with BRIG using pMCR_1511 (KX377410) as reference. The 2 inner rings reveal GC content and GC skew and the outer ring represents the coding sequences (CDS) of reference. Unlabeled CDS represents genes encoding hypothetical proteins

The two IncP-1 GenBank sequences originated from mainland China. The 57,278-bp plasmid pMCR_1511 (KX377410) was isolated from *Klebsiella pneumoniae* and the 47,824-bp plasmid pMCR16_P053 (KY352406) was identified in *Salmonella enterica* subsp. *enterica*. pMCR_1511 was used as reference for comparative sequence analysis (Fig. 3). The IncP-1 plasmid of this study was very similar to pMCR16_P053 (99% sequence homology), while both of them were different from reference by the absence of 9 genes and insertion sequences, including *bla*_TEM_ (non-ESBL), *ble* (bleomycin resistance)*, dgkA* (diacylglycerol kinase)*, eptA* (phosphoethanolamine transferase)*, higA* (anti-toxin), *higB-1* (probable mRNA interferase), *ybaQ* (transcriptional regulator), IS26 and ISApl1.

**Fig. 4:**
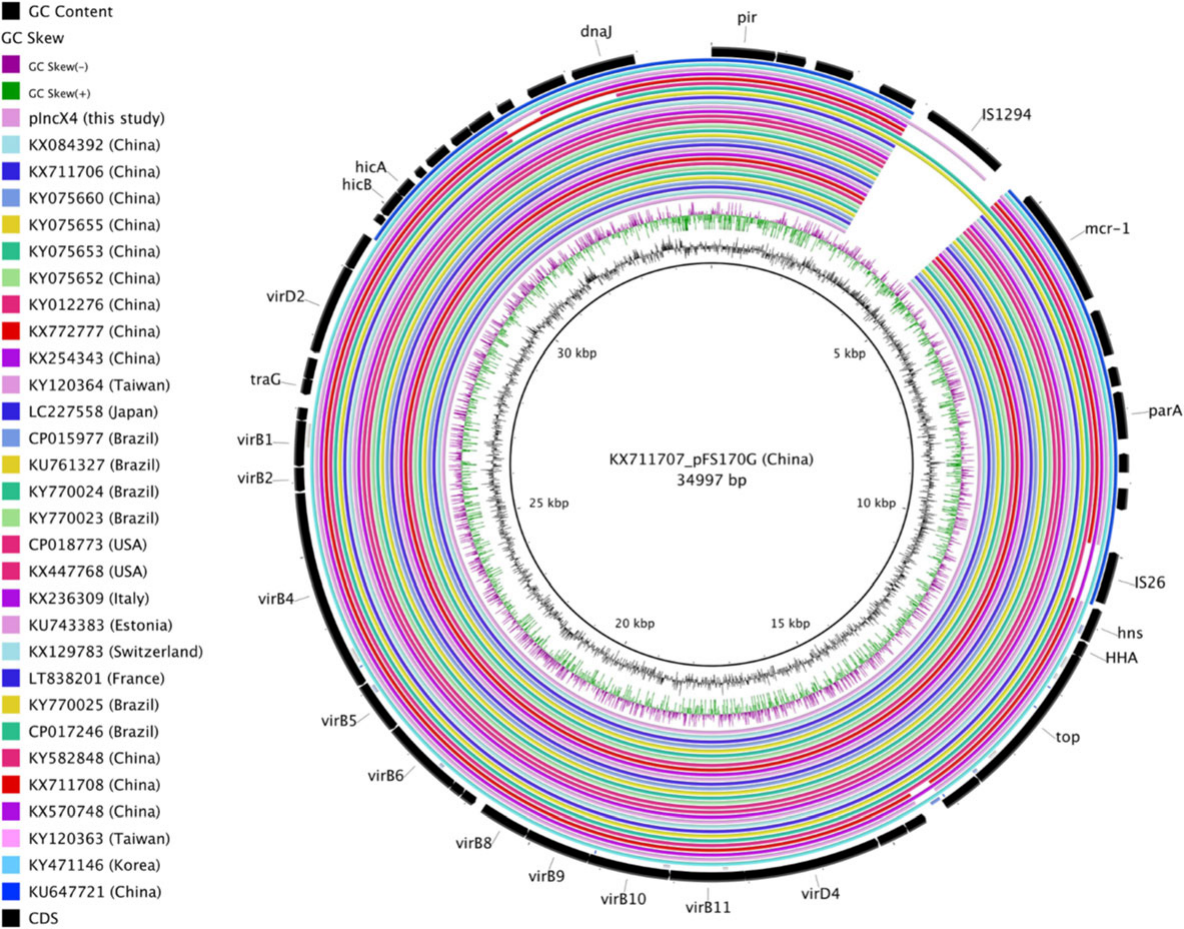
Comparative sequence analysis of IncX4 type of *mcr-1* plasmids. The circular image was generated with BRIG using pFS170G (KX711707) as reference. The 2 inner rings reveal GC content and GC skew and the outer ring represents the CDS of reference. Unlabeled CDS represents genes encoding hypothetical proteins

The 30 IncX4 GenBank sequences were diversified in geographical origins (from 10 regions, with 18 entries from Asia, 8 from America and 4 from Europe). There were 2 ranges of plasmid sizes, with 27 sequences between 31,229 and 34,997 bp (< 40,000 bp) and 3 sequences from 42,941 to 48,350 bp (> 40,000 bp). The hosts of these plasmids included *E. coli* (86.7%), *K. pneumoniae* (6.7%) and *S. enterica* subsp. *enterica* (6.7%). Plasmid pFS170G (KX711707) was used as reference for comparative sequence analysis (Fig. 4). Majority of these sequences were very similar, and the IncX4 plasmid of this study shared highest homology (99%) with pICBEC3AM (KY770024). IS26 was the predominant type of insertion sequence (78.6%). Carbapenemase/ ESBL genes were absent in most of these plasmids (96.8%), while pCQ02-121 (KU647721) was the only plasmid harboring carbapenemase gene (*bla*_NDM-5_). In fact, it was an IncX3-IncX4 hybrid plasmid identified in an *E. coli* isolate [39], which might account for its difference from majority.

#### Implications from coexistence of IncP-1 and IncX4 plasmids

Coexistence of IncP-1 and IncX4 types of *mcr-1* plasmids in the same *E. coli* isolate was indeed a combination of stability and high transmissibility - the broad-host-range IncP-1 plasmids can transfer to, replicate in and stably maintained in virtually all species of Gram-negative bacteria [40, 41], while IncX4 plasmids are self-transferable at high frequency (~10^-1^ to ~10^-4^) which have contributed to intercontinental spread of *mcr-1* genes [42]. These 2 incompatibility groups of *mcr-1* plasmids could be harbored by at least *E. coli, K. pneumoniae* and *S. enterica* subsp. *enterica,* and the list is expected to be expanding. Another aspect of concern is the potential of IncP-1 and IncX4 plasmids to acquire a wide spectrum of antibiotic resistance genes, for instance, ESBL and carbapenemase genes [39, 43–45], which may contribute to pan-drug resistance under selective pressure [46, 47]. Nevertheless, our study was not the sole report on *Enterobacteriaceae* harboring more than 1 copy of *mcr-1* gene [42, 48]. Interestingly, colistin MICs of these isolates appeared to be unaffected by gain of extra copies of *mcr-1* gene, which was coherent with the finding in our study. While the rationale behind this phenomenon remains obscure, it may reflect the active nature of transposons carrying *mcr-1* gene, for instance, ISApl1 transposons [49]. The mechanism behind mobilization of *mcr-1* genes and its integration into a variety of plasmids warrant further investigation.

#### Limitations of this study

Our study had several limitations. First, we presented the surveillance data of a regional hospital. Further study with larger cohort of experimental subjects from representative geographical locations is warranted to have a more complete picture on prevalence and characteristics of *mcr-*possessing *Enterobacteriaceae* in Hong Kong. Second, we could not assess any information regarding antibiotic usage by experimental subjects outside our hospital. In addition, albeit not within the scope of this study, further curation and analysis of WGS data can provide insights into actual genomic locations of *mcr-1* in remainder *E. coli* isolates.

## Conclusions

In this study, we have further revealed asymptomatic carriage of *mcr-1-*harboring *Enterobacteriaceae* by both patients and healthy individuals, with fecal carriage of 2.08% which was higher than expected in our region. This asymptomatic carriage is alarming because it potentially facilitates acquisition of *mcr-1* by multidrug-resistant bacteria in future infection, which severely limits the choice of usable antibiotics for treatment. Prevalence of *mcr-2-*harboring *Enterobacteriaceae*, on the contrary, is believed to be very low at present. This surveillance data on emerging antibiotic resistance is important for infection control, especially for patients under intensive care. In addition, we have unraveled the phenotypic and molecular traits of 14 *mcr-1-*possessing *E. coli* strains. Further studies on mobilization and resistance mechanisms of this recently-discovered resistance gene family may shed light on strategies limiting emergence and spread of pan-drug-resistant bacteria.

## Abbreviations

AM: Ampicillin
AMC: Amoxicillin-clavulanate
AN: Amikacin
BMD: Broth microdilution
BRIG: BLAST Ring Image Generator
CAZ: Ceftazidime
CDS: Coding sequence
CF: Cephalothin
CIP: Ciprofloxacin
CLSI: Clinical & Laboratory Standards Institute
CN: Gentamicin
CTX: Cefotaxime
CXM: Cefuroxime
ESBL: Extended-spectrum β-lactamase
EUCAST: The European Committee on Antimicrobial Susceptibility Testing;
FEP: Cefepime
HA: Clients coming for health assessment
hp: Hypothetical protein
I: Intermediate
IP: Inpatients
LEV: Levofloxacin
MALDI-TOF MS: Matrix-assisted laser desorption/ionization-time of flight mass spectrometry
*mcr-1* and *mcr-2*: Mobilized colistin resistance genes
MI: Minocycline
MIC: Minimum inhibitory concentration
OP: Outpatients
PB: Polymyxin B
PCR: Polymerase chain reaction
PE: Colistin
R: Resistant
ST: Sequence type
SXT: Trimethoprim/sulfamethoxazole
TIM: Ticarcillin-clavulanate
TOB: Tobramycin
TZP: Piperacillin-tazobactam
VRE: vancomycin-resistant enterococci
WGS: Whole genome sequencing
